# Epigenetic disruption meets immune deficiency: a case report of ICF syndrome linked to DNMT3B mutation

**DOI:** 10.3389/fimmu.2025.1742293

**Published:** 2026-01-21

**Authors:** Zaid Al Ali, Khaled F. Al-Ali, Fatima Bader, Nancy Alhalabiya, Deema Sous, Ayah Abulehia

**Affiliations:** 1 Medical Research Club, Faculty of Medicine, Al-Quds University, Jerusalem, Palestine; 2 Department of pediatrics, An-Najah National University, Ramallah, Palestine

**Keywords:** case report, centromeric instability, facial dysmorphism syndrome, ICF syndrome, primary immunodeficiency

## Abstract

Immunodeficiency, centromeric instability, and facial anomalies (ICF) syndrome is a rare, autosomal recessive primary immunodeficiency, with fewer than 120 cases reported worldwide. ICF type 1 (ICF1) is the most prevalent subtype. Despite its rarity, ICF1 presents a distinct set of clinical features that necessitate increased awareness, particularly in populations with high rates of consanguinity. This case presents a two-year-old Palestinian boy born to consanguineous parents who presented with recurrent respiratory tract infections, facial dysmorphisms, and hypogammaglobulinemia. A comprehensive immunologic evaluation confirmed markedly reduced immunoglobulin levels consistent with an antibody deficiency. Genetic testing identified a homozygous missense mutation in *DNMT3B* (Arg826Cys), establishing the diagnosis. The patient was started on intravenous immunoglobulin (IVIG) replacement therapy, which was well tolerated and led to a noticeable reduction in infection frequency and an overall clinical well-being improvement. While treatment remains supportive, early recognition and immunologic management can significantly reduce morbidity. This case highlights the importance of early diagnosis and immunologic support in ICF1 syndrome, reinforces genotype–phenotype correlations, and provides valuable insights from an underrepresented region to improve global awareness, diagnosis, and care, being only the second genetically confirmed case from Palestine.

## Introduction

ICF syndrome is an autosomal recessive primary immunodeficiency that has been documented in approximately 120 patients worldwide (1, 2). It is characterized by profound immune system dysfunction due to hypogammaglobulinemia, often resulting in recurrent respiratory and gastrointestinal infections (2, 3). Additionally, patients present with delayed developmental milestones with various degrees of cognitive impairment and characteristic facial dysmorphisms, such as hypertelorism, epicanthic folds, low-positioned ears, and a flattened nasal bridge (3).

ICF syndrome is classified into five genetically different subtypes (4). ICF1, the most common, accounts for about 60% of cases and is caused by mutations in the DNMT3B gene. ICF2, representing about 30% of cases, is associated with pathogenic variants in ZBTB24. Less commonly, ICF3 and ICF4 are linked to mutations in CDCA7 and HELLS, respectively. A minority of patients lacking identifiable genetic alterations are categorized under a provisional subtype known as ICFX (4).

The DNMT3B (DNA methyltransferase 3B) gene plays a crucial role in the *de novo* DNA methylation during embryonic development, particularly at the centromeric, pericentromeric, and subtelomeric regions. This methylation maintains accurate transcription and genomic stability. However, in cases of ICF1 syndrome, mutations in the DNMT3B gene impair its catalytic activity, leading to widespread DNA hypomethylation, causing chromosomal instability at chromosomes 1, 9, and 16. In addition to its role in DNA methylation, the DNMT3B gene also regulates the balance between sense, antisense gene expression and alternative splicing. For example, the failure to suppress the antisense transcript of CD27 results in its downregulation, which decreases the formation of memory B cells and adversely affects long-term immune defense (5).

In the Middle East, there have been twelve reported cases of ICF syndrome: four in Lebanon, five in Saudi Arabia, one in Palestine, and two in Iran (6). In conclusion, despite the typical presentation of the syndrome, its rarity necessitates a high level of suspicion. Reporting genetically confirmed cases, particularly from regions with high rates of consanguinity, strengthens genotype–phenotype correlations and raises awareness, aiding in earlier recognition and management of the condition.

## Clinical presentation

A male infant, born at term (38 weeks’ gestation) via an uncomplicated vaginal delivery, with a birth weight of 2800 grams. At four months of age, the patient’s mother first noticed feeding intolerance and poor weight gain. Shortly after receiving routine vaccinations at four months, he developed poor feeding and lethargy, which required intravenous fluids for dehydration. Between four and eight months of age, he experienced recurrent infections, some severe enough to require hospitalization. During this time, he also had repeated episodes of otitis media with prolonged unilateral ear discharge, prompting multiple evaluations by the otolaryngology team. Because of the frequency and severity of these infections, an immunological evaluation was performed at eight months of age, revealing significant hypogammaglobulinemia, as shown in **Table 1**. As a result, IVIG therapy was initiated at that time.

**Table 1:** Changes in immunoglobulin (Ig) levels according to the patient’s age.

Ig type	7 months	10 months	11 months	16 months	18 months	26 months
IgA	<5 mg/dl*	10 mg/dl	NR	8 mg/dl	3 mg/dl	NR
IgG	21 mg/dl*	697 mg/dl	672 mg/dl	763 mg/dl	751 mg/dl	1085 mg/dl
IgM	1 mg/dl*	15 mg/dl	NR	13 mg/dl	15 mg/dl	NR

*Reference range: IgG (mg/dl): 5-<9 months: 164-588; 9-<15 months: 246-904; 15-<24 months: 313-1,170. IgA (mg/dl): 5-<9 months: 16-50; 9-<15 months: 27-66; 15-<24 months: 36-79. IgM (mg/dl): 5-<9 months: 32-132; 9-<15 months: 40-143; 15-<24 months: 46-152.

Physical examination revealed dysmorphic features, including epicanthic folds, frontal bossing, and a flat nasal bridge. Oral examination demonstrated gingival swelling and absent upper incisors. Developmental concerns were evident, with global developmental delay noted. In view of the recurrent infections, immunodeficiency, dysmorphic features, and developmental delay, genetic testing was pursued at eight months of age. Whole-exome sequencing identified a homozygous DNMT3B (Arg826Cys) mutation, confirming the diagnosis of ICF syndrome type 1. Therefore, The plan is to continue IVIG therapy monthly at a dose of 400 mg/kg. Both parents were first cousins and heterozygous carriers of the same mutation.

At two years of age, while receiving regular IVIG therapy, the patient was admitted with a breakthrough febrile illness characterized by a four-day history of high-grade fever (up to 39°C), tachypnea, reduced oral intake, decreased activity, unilateral ear discharge, and two episodes of non-bilious, non-bloody vomiting. On examination, he appeared mildly dehydrated but was alert and interactive. Vital signs revealed an oxygen saturation of 97%, heart rate of 136 beats per minute, respiratory rate of 46 breaths per minute, and blood pressure of 85/60 mmHg. Laboratory investigations demonstrated a markedly elevated C-reactive protein level (98 mg/L), while blood, stool, and urine cultures were negative. In light of his clinical presentation and history of recurrent infections, a sepsis workup was undertaken. He was treated empirically with intravenous piperacillin–tazobactam and oral acyclovir for suspected viral gingivostomatitis, in addition to intravenous fluids for dehydration and his scheduled IVIG infusion.

Developmentally, the child continued to exhibit global delays. He achieved independent walking at approximately two years of age and, at 26 months, had not yet developed meaningful words. His developmental level was assessed to be consistent with that of a 9-10-month-old infant. Audiologic evaluation remains inconclusive, with auditory brainstem response testing pending. Neurological examination revealed no meningeal signs; however, cranial assessment raised concern for possible increased intracranial pressure, prompting referral to neurosurgery.

Following initiation of regular IVIG therapy, his growth trajectory gradually improved, with subsequent catch-up growth; his current weight is 12 kg He continues to receive monthly IVIG at a dose of 400 mg/kg and is regularly followed in immunology, audiology, and speech therapy clinics. Immunoglobulin levels are shown in **Table 1**, and a brief overview of the patient’s clinical characteristics, investigations, and outcomes is in **Table 2**.

**Table 2:** Summary of clinical characteristics, investigations, and health outcome of ICF syndrome patient.

Age/timepoint	Key events	Management/outcome
4 months	Feeding intolerance and poor weight gain noticed by mother; lethargy, poor feeding after routine vaccination	IV fluids for dehydration; stabilized
4–8 months	Recurrent infections,including otitis media	Supportive care; hospitalizations as needed
8 months	Persistent infections, developmental delay, dysmorphic features; hypogammaglobulinemia; genetic testing confirmed immunodeficiency, centromeric instability, and facial anomalies (ICF) syndrome type 1	Monthly IVIG (400 mg/kg) initiated; ongoing therapy
2 years	Breakthrough febrile illness on IVIG: fever, reduced intake, unilateral ear discharge, vomiting; CRP elevated, cultures negative	Empiric IV antibiotics and oral acyclovir; IV fluids; continued IVIG; improved
Current	Developmental delays; growth monitoring	Continued monthly IVIG; follow-up in immunology, audiology, and speech therapy; gradual catch-up growth (now 12kg)

## Patient perspective

The patient’s mother expressed great concern during her child’s early progress. This was mainly due to recurring infections, feeding issues, and delayed development without a clear diagnosis. She felt relieved when the immunologic evaluation identified the underlying condition and started IVIG therapy. She has seen noticeable improvement in her child’s health and is thankful for finally understanding the cause of his symptoms. The family is dedicated to ongoing follow-up and supportive care.

## Discussion

ICF syndrome is still one of the rarest autosomal recessive primary immunodeficiencies, with fewer than 120 cases documented around the world. It comprises five genetically distinct subtypes, with ICF1 (caused by DNMT3B mutations) being the most common (4, 6). This case presents a two-year-old male with ICF syndrome type 1, confirmed genetically by a homozygous mutation in DNMT3B Arg826Cys. The clinical presentation of the child lines up with the classical features of ICF syndrome: dysmorphic facial characteristics (hypertelorism, epicanthal folds, flat nasal bridge, and low-set ears), recurrent infection, and severe hypogammaglobulinemia (7, 8).

The DNMT3B gene encodes a DNA methyltransferase that is important for *de novo* adding methyl groups to CpG during embryogenesis, especially in heterochromatic areas. When DNMT3B loses its function, it stops methylation at satellite DNA sequences, especially on chromosomes 1, 9, and 16. This process makes the chromosomes unstable, which is a key sign of ICF1 syndrome (5). In addition, impaired methylation will consequently affect the expression of numerous genes, including those essential for immune response and development. DNMT3B deficiency impairs CD27 expression by failing to silence antisense CD27 transcripts, which affects the formation of memory B cells and leads to the patient’s hypogammaglobulinemia and recurrent infections (5, 9).

Several reports indicate that ICF syndrome may present with minimal dysmorphic features, making early recognition challenging. For example, a 2018 study investigating children with persistent hypogammaglobulinemia identified DNMT3B mutations in patients who exhibited only mild facial dysmorphism and cytogenetic abnormalities (10). Furthermore, a 2019 case report detailed a child with a previously unreported DNMT3B variant who demonstrated significant centromeric instability on cytogenetic testing, despite an initial clinical evaluation that did not strongly indicate ICF syndrome (11). These findings collectively emphasize the necessity for a thorough assessment of unexplained hypogammaglobulinemia. Incorporating chromosomal analysis alongside targeted genetic testing can help ensure that ICF syndrome is considered and not overlooked in children who lack the more classical phenotypic features.

Published literature documents only a limited number of ICF syndrome cases from the Middle East. The most recent report by Joma et al. (2025) described a Palestinian child with recurrent infections, hypogammaglobulinemia, and craniofacial anomalies, in whom whole-exome sequencing identified a novel homozygous DNMT3B variant (p.Arg826Cys) within the catalytic domain (6). Reporting additional genetically confirmed cases from this region is important for improving recognition of ICF syndrome, particularly in populations with high rates of consanguinity. Such contributions help refine genotype–phenotype correlations and support earlier consideration of ICF syndrome in the evaluation of unexplained hypogammaglobulinemia.

Treatment of ICF syndrome primarily relies on IVIG replacement therapy, often supplemented with prophylactic antibiotics to reduce infectious complications (12). IVIG helps correct the underlying antibody deficiency and lowers the frequency and severity of recurrent infections. Reported survival rates with IVIG-based supportive care range from 60% to 84%, reflecting its clinical effectiveness. Despite this, some patients continue to experience severe or life-threatening disease due to persistent infections, immune dysregulation, or hematologic malignancies. For individuals with profound immunodeficiency, allogeneic hematopoietic stem cell transplantation (HSCT) remains the only intervention with curative potential (12).

For this patient, IVIG replacement therapy was initiated in response to markedly reduced immunoglobulin levels identified at 8 months of age. He received a fixed monthly dose of IVIG (approximately 400 mg/kg for a 12-kg child), administered intravenously over 8 hours with standard premedication. A two-week delay in accessing care required a compensatory half-dose (2.5 g) to maintain therapeutic coverage. No prophylactic antibiotics were given during this period.

Following initiation of IVIG therapy, IgG levels increased rapidly, from 21 mg/dL at 7 months to 697 mg/dL at 10 months, and remained stable within the normal range for age (751 mg/dL at 18 months and 1085 mg/dL at 26 months), providing effective IgG-mediated protection. IgA and IgM levels, however, remained persistently low, consistent with the B-cell maturation defect characteristic of ICF syndrome and reflecting the ongoing underlying immunodeficiency. Although routine monitoring of IgA and IgM was limited by financial constraints, the clinical response was evident, with a clear reduction in the frequency and severity of infections as reported by the parents and noted during monthly follow-up visits. These findings support the effectiveness of IVIG therapy in improving humoral immunity in ICF syndrome, even without adjunctive antibiotic prophylaxis.

This clinical course illustrates both the therapeutic value and the inherent limitations of IVIG in ICF syndrome. While IVIG effectively reduces infection burden, it does not correct the underlying genetic and epigenetic defects. Consequently, long-term multidisciplinary follow-up remains essential, including continued neurodevelopmental monitoring given the variability in cognitive outcomes associated with ICF1 (13).

This case illustrates that a thorough diagnostic process with regular follow-ups can lead to a clear diagnosis and an effective treatment plan. The identification of a homozygous DNMT3B variant was a significant finding in the diagnosis, while ongoing monitoring of IgG levels and infection history provided a reliable measure of the patient’s response to therapy. However, important limitations persist. Financial and logistical barriers prevented us from measuring IgA and IgM levels, conducting detailed B-cell studies, or confirming centromeric instability, which is a crucial diagnostic feature of classic ICF1. Additionally, interruptions in care, such as delayed administration of IVIG, may have affected the patient’s clinical course.

Furthermore, this case supports existing knowledge about ICF1 while offering new insights. The child’s symptoms and test results align with those observed in similar cases, reinforcing the internal validity of our findings. However, variations in physical features and a strong early response to IVIG demonstrate that each case can be unique. This case highlights the unpredictable nature of ICF1 and emphasizes the importance of sharing experiences from different contexts. Such data helps build a clearer understanding of how the disease presents itself, thereby contributing to external validity.

## Conclusion

In conclusion, this report broadens the clinical and genetic characterization of ICF syndrome within an underrepresented region. The confirmed DNMT3B mutation and associated clinical features contribute to strengthening genotype–phenotype correlations. Early diagnosis and timely immunologic intervention remain central to reducing morbidity and informing long-term management. Continued reporting of genetically confirmed cases, supported by expanding molecular diagnostic capabilities, will advance knowledge of disease heterogeneity and improve patient outcomes worldwide.

## Data Availability

The original contributions presented in the study are included in the article/supplementary material. Further inquiries can be directed to the corresponding authors.
